# Inadequate Utilization of Prenatal Care Services, Socioeconomic Status, and Educational Attainment Are Associated with Low Birth Weight in Zimbabwe

**DOI:** 10.3389/fpubh.2017.00035

**Published:** 2017-03-06

**Authors:** Sanni Yaya, Ghose Bishwajit, Michael Ekholuenetale, Vaibhav Shah

**Affiliations:** ^1^Faculty of Social Sciences, School of International Development and Global Studies, University of Ottawa, Ottawa, ON, Canada; ^2^School of Medicine and Health Management, Tongji Medical College, Huazhong University of Science and Technology, Wuhan, China; ^3^The Women’s Health and Action Research Centre, Benin City, Nigeria; ^4^Interdisciplinary School Health Sciences, University of Ottawa, Ottawa, ON, Canada

**Keywords:** prenatal care, low birth weight, neonatal and infant mortality, global health, Zimbabwe

## Abstract

**Background:**

Globally, low birth weight (LBW) remains a leading cause of neonatal and infant mortality and poses significant challenges toward the progress of achieving infant mortality-related goals. Experience from developed countries shows that two major causes of LBW (premature delivery and intrauterine growth restriction) can be averted to a great extent by adequate utilization of maternal health-care services, during pregnancy. In this study, we attempt to measure the prevalence of LBW in Zimbabwe and explore the association between adequate utilization of prenatal care (PNC) services and LBW in Zimbabwe. We also explore other possible associations with LBW.

**Methodology:**

This study was based on nationally representative, cross-sectional data from Multiple Indicator Cluster Survey round 5, conducted in 2014. Participants included 3,221 mothers from both rural and urban areas. The participants were selected regardless of their current pregnancy status. Sample characteristics were presented using descriptive statistics. Association between utilization status of ANC and LBW was measured by chi-square (bivariate) test and logistic regression methods.

**Results:**

Prevalence of LBW was 12.8%. There was 11% reduction in the odds of having LBW babies for participants from urban area when compared with rural area (AOR = 0.897; 95% CI = 0.707–1.138). When compared to women with higher education, those having primary/below primary and secondary level qualification had higher odds of experiencing LBW babies by 73 and 56%, respectively. Participants who had less than four PNC/ANC visits had 34% higher odds (AOR = 1.340; 95% CI = 1.065–1.685) than those with at least four visits, and those who had given birth more than once, had 38% lower odds (AOR = 0.620; 95% CI = 0.493–0.780) of giving birth to LBW babies when compared to those who had given birth only once.

**Conclusion:**

The findings of this study have programmatic and policy implications for low-resource nations and suggest that promoting access to ANC services especially in the rural areas is likely to reduce prevalence of LBW in Zimbabwe. This is important as LBW babies consume lot of health resources *per se* and not only in terms of hospitalization but also in terms of outpatient and physician visits during the first year of their life.

## Introduction

Low birth weight (LBW) is regarded as an important predictor of public health and a measure of progress toward sustainable development goals (SDGs) in developing countries (1). The SDGs, prominently called Transforming our world: the 2030 Agenda for Sustainable Development, entails 17 aspirational “Global Goals” with about 169 targets between them. It is led by the United Nations, through a deliberative process involving its 194 Member States with global civil society. World Health Organization (WHO) has set a threshold for LBW for international comparison at a birth weight of less than 2.5 kg (5.5 lb) (2). Studies have found that LBW babies are about 20 times more likely to die in infancy compared to normal birth weight (NBW) babies, and those who survive, share a greater burden of various physical (3) and psychological complications, such as behavioral and cognitive disorders. The resulting health-care expenditures are also higher for the surviving LBW babies (4).

In 2003, about 17% of infants in developing countries were born with LBW. Though there were disparities in the prevalence of LBW within countries, on the average, about 13% of births in sub-Saharan Africa (SSA) were categorized as LBW (5). Apart from health consequences for the individual, LBW baby can influence the family planning decisions, future expectations or desire for more children, and status of the mother in the family and can lead to elevated levels of stress among parents. Previous studies have found that mothers of infants born preterm or LBW (PT/LBW) are at risk for experiencing psychological distress and depression following the child’s birth (6). LBW cases can translate into deeper social and economic consequences besides direct health impacts. Given the critical nature of the issue, the comprehensive implementation plan on maternal, infant, and young child nutrition endorsed by World Health Assembly Resolution is committed to a target of 30% reduction of LBW babies by the year 2025.

Also, previous studies have attempted to explore the impact of maternal health-care services (MHS) utilization on birth weight outcomes. A growing body of evidence suggests that utilization of prenatal care (PNC) services holds a great potential in improving pregnancy outcomes. Intuitively, maternal health status and access to MHS are two very crucial indicators of child health. However, the utilization of PNC services remains remarkably low in the countries of the SSA region, including Zimbabwe (7). Studies on maternal level determinants, such as health and socioeconomic factors, in relation to LBW have important significance for health policy making since such investigations provide workable insights about the risks factors of LBW (8, 9).

Preterm birth and intrauterine growth retardation (IUGR) are cited as the two main clinical factors behind LBW. Much of the information on LBW is hospital based, and there is dearth of information on community-based factors (10). Most of the efforts to reduce LBW have remained unrealized at best, even in developed countries like USA (11). Accordingly, the pertinent question is which socio-demographic variables are associated with LBW? And can these associated variables be manipulated to reduce the incidence of LBW? Answering these questions might help us influence the policy dynamics behind LBW (12).

There are several variables when it comes to confounding effects on a dependent variable like LBW. For example, one study found that 90 LBW infants per 1,000 live births were born from 1975 through 1979 in South Carolina. In this study, after controlling for the confounding variables like education and wealth inequity, presence of Black ancestry was found as an independent variable affecting birth weight of the infant (13). Similarly, another study also reported higher incidence of IUGR in black, single, primiparous, women of age <17 or >30 years with history of preterm delivery, short, thin, and those who consumed alcohol or abused drugs (14). Another study found housing conditons as an independent variable affecting LBW. This was apart from the associated confounding factors like poor PNC, previous history of preterm birth, and low maternal body mass index (15).

In addition, one study reported several risk factors associated with the presence of LBW. However, socioeconomic status was associated with only extreme cases of LBW (16–18). It has been found in a study that although most socioeconmic factors are associated with pregnancy outcomes, the pattern of association is clear only for LBW (19). This raises a crucial point; of the many factors cited as impacting LBW, which factors should be focused on or, in other words, are promising to get measurable and reasonable results, meeting the WHO target of 30% reduction in LBW by 2025? This study accordingly aims to study those factors, which are promising for reducing LBW as well as can have wider ramifications (20). Another study claims that cigarette smoking and bacterial vaginosis are factors, which explain the socioeconomic disparities in IUGR cases. This further potentiates the study of socioeconomic disparities which remain amenable to policy actions and accordingly make LBW as an amenable entity through mediation of the vicious cycle of socioeconomic disparities, predisposing factors of IUGR, and the prevalence of infant morbidity and mortality through IUGR (21, 22). The goal of the present study was to explore the maternal determinants in the country based on a nationally representative data from Multiple Indicator Cluster Survey (MICS) survey.

## Materials and Methods

### About the Survey Program and Data Collection

The MICS program was launched in the mid-1990s with the aim to provide quality data about individuals (women and children) and households, on a wide range of socioeconomic and health indicators. Information collected is internationally comparable and range from crucial topics, such as malaria, HIV, health knowledge, and health service utilization. Surveys are designed, based on the assessed priorities of data requirements at national and subnational levels. In conjunction with UNICEF, the MICS program is currently operational in 109 countries and contributes to policy making toward and promotion of maternal and child health by providing data sources in the given countries. UNICEF Regional Office provides technical support for the MICS program.

### Sampling of Study Population

Using 2012 census data as the sample frame, census enumeration areas, being used as primary sampling units, were defined and selected from the two strata, urban and rural created from each of the nine provinces of Zimbabwe except one province, the tenth province of Zimbabwe, Bulawayo, which had no stratification of urban and rural areas. This was the first stage of sampling in which specified number of clusters, a total of 683, was selected with probability proportional to size. The number of households in the clusters determined the size. In the second stage, a list of households was separately created through field visits owing to the inadequacy of census listing of households. A total of 25 households were selected in each cluster resulting in a total selection of 17,075 households. One cluster in Masvingo province was not enumerated due to flooding and relocation of the households.

The survey included four types of questionnaires: one for the households, one for women aged 15–49 years, one for men aged 15–59 years, and one for children aged below 5 years. For this study, we utilized women sample dataset to gather information on LBW and the related variables. From the selected households, 12,507 women were identified for interview, and finally, 11,510 were successfully interviewed with a response rate of 92%. Face-to-face interviews were conducted for all women aged 15–49 years in the selected households and men aged 15–59 years in every third household from the sampled households, by use of questionnaires covering socioeconomic, demographic, and health indicators. Overall response rate was 98%.

### Variables

The outcome variable was LBW and was defined as birth weights less than 2.5 kg. Teenage pregnancy was defined as pregnancy with age of mother less than 19 years at the time of the survey.

### Data Analysis

The baseline characteristics of the sample population were presented by descriptive statistics (numbers and percentages). Prevalence of LBW across the explanatory variables was presented in numbers, and percentages and the difference between two groups (LBW, NBW) were estimated by Chi-square bivariate tests. The explanatory variables, which showed significant associations with LBW, were entered in the regression model (Generalized estimating equations).

Regression analysis was then carried out to determine the strength of association between the exposure variables and the outcome, LBW. Results of regression were reported in terms of odds ratios and 95% confidence interval. *p*-Value of <0.05 (two-tailed) was considered statistically significant. All analyses were performed with SPSS^®^ 21 for Mac.

### Ethical Approval

Data used in this study are secondary, available in public domain, and were obtained through registration in the MICS website. Furthermore, institutional approval was not necessary since UNICEF who approved the data for this research has the data available under public domain. More details regarding the MICS data and ethical standards are available at: http://mics.unicef.org/surveys.

## Results

### Descriptive Statistics

**Figure 1:**
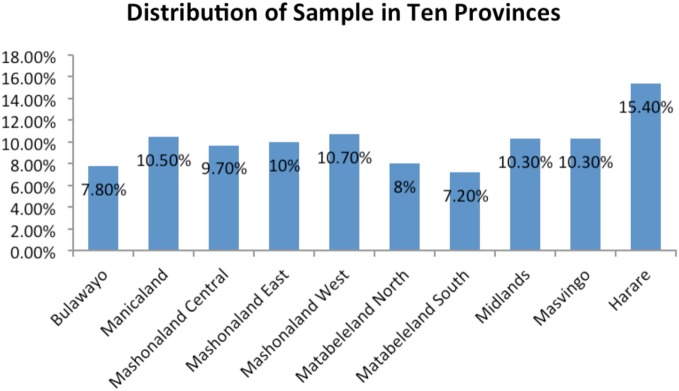
**Distribution of the samples in 10 provinces**.

**Figure 2:**
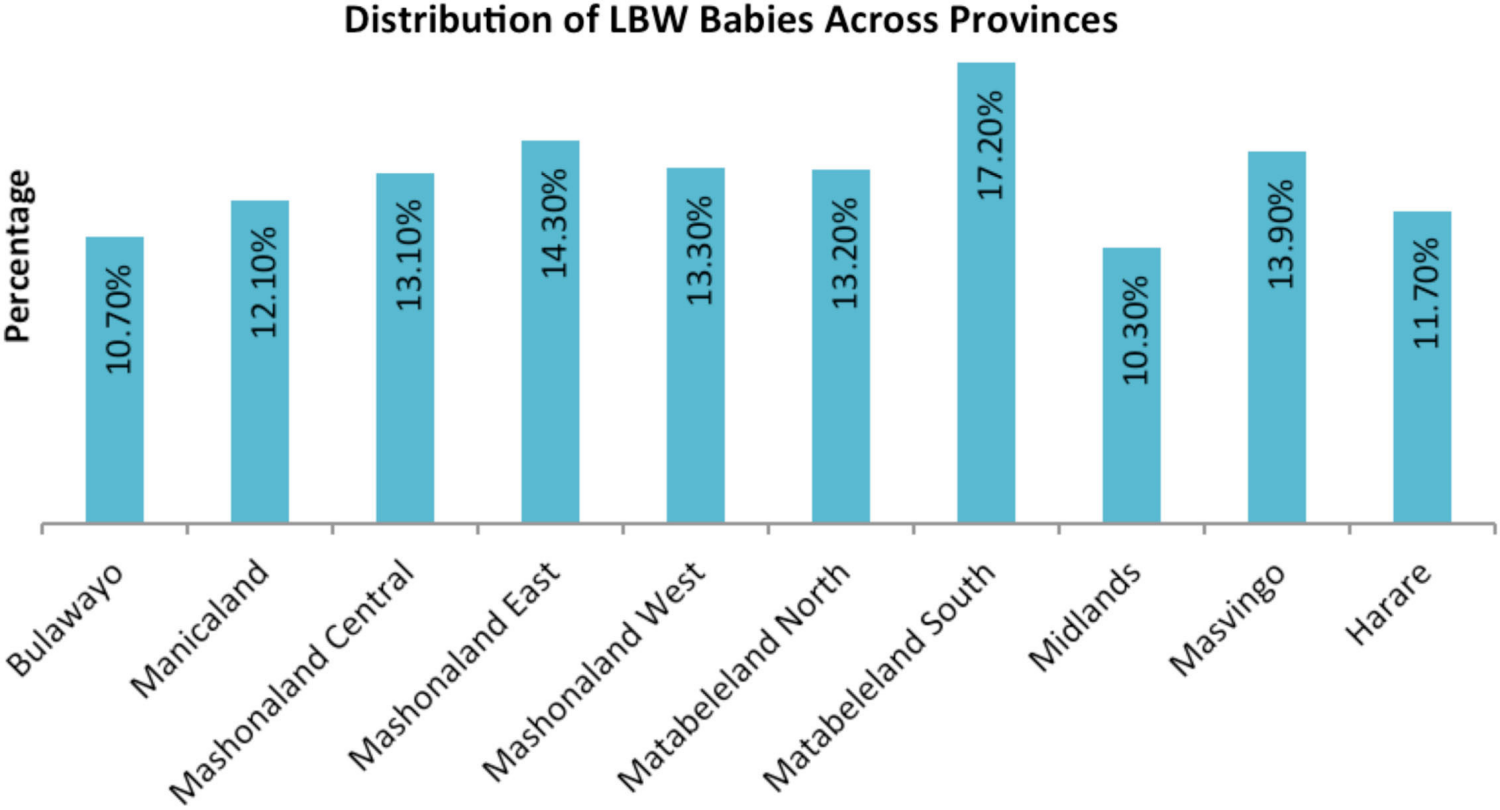
**Distribution of low birth weight (LBW) babies across provinces**.

The sample included 3,221 women in the 15–49 years age group. The prevalence of LBW babies was 12.8% at national level. Province-wise distribution of sample population and LBW babies are shown in Figures 1 and 2, respectively. The mean LBW (2.213 ± 0.367 kg) and the mean NBW (3.263 ± 0.431 kg) groups were calculated.

Table 1 contains the baseline characteristics of the participants. The table shows that almost two-third of the population resided in rural areas (64.2%). With a statistically significant difference, 13.5% of those from the rural areas had LBW babies compared to 11.6% of those from the urban areas. About one-fourth (26.9%) had primary/under primary and two-third (66.6%) had secondary level schooling experience. Only 6.6% had higher than secondary level of schooling. Of those with primary/under primary, secondary, and higher than secondary level of schooling, 13.9, 12.9, and 8.1% had LBW babies, respectively, and the differences were statistically significant with *p* = 0.036. Regarding wealth status, more than half of the women reported residing in poor economic conditions (51.7%). Of these, 14% had LBW babies compared to 11.6% of those from non-poor background, and the difference was statistically significant. A higher percentage of women reported having ever drunk alcohol as compared to those having ever smoked. However, both factors were found statistically not significant for the occurrence of LBW babies. About one-third of women reported last pregnancy as unplanned (30.9%), and 33.5% reported becoming pregnant before reaching 18 years. Only about a quarter (23.7%) of the women received at least four prenatal/antenatal visits and 70.2% were primiparous and both these variables were statistically significant associated with the occurrence of LBW babies.

**Table 1:** **Basic characteristics of the study population (*n* = 3,221)**.

Variables	Operational definitions	*N* (%)	Birth weight		*p*-Value
			Low birth weight (LBW)	Normal birth weight	
**Residency**					
Urban	Area of residency of participants	1,154 (35.8)	134 (11.6)	1,020 (88.4)	0.049
Rural		2,067 (64.2)	279 (13.5)	1,788 (86.5)	
**Educational attainment**					
Under primary/primary	Level of formal schooling experience	865 (26.9)	120 (13.9)	745 (86.1)	0.036
Secondary		2,145 (66.6)	276 (12.9)	1,869 (87.1)	
Higher		211 (6.6)	17 (8.1)	194 (91.9)	
**Wealth status**					
Poor	Overall economic status of the household	1,665 (51.7)	233 (14)	1,432 (86)	0.022
Non-poor		1,556 (48.3)	180 (11.6)	1,376 (88.4)	
**Ever smoked**					
Yes	Whether or not participant tried smoking cigars	51 (1.6)	7 (13.7)	44 (86.3)	0.912
No		3,169 (98.4)	406 (12.8)	2,763 (87.2)	
**Ever drank alcohol**					
Yes	Whether or not participant tried alcoholic drinks	488 (15.2)	57 (11.5)	432 (88.5)	0.563
No		2,728 (84.7)	356 (13)	2,372 (87)	
**Last pregnancy wanted**					
Yes	Whether or not last pregnancy was planned	2,225 (69.1)	285 (12.8)	1,940 (87.2)	0.507
No		996 (30.9)	128 (12.9)	868 (87.1)	
**Teenage pregnancy**					
Yes	Became pregnant before reaching 18 years	1,079 (33.5)	136 (12.6)	943 (87.4)	0.420
No		2,142 (66.5)	277 (12.9)	1,865 (87.1)	
**Prenatal care (PNC)**					
4 Visits	Times received PNC	764 (23.7)	119 (15.6)	645 (84.4)	0.006
<4 Visits		2,457 (76.3)	294 (12)	2,163 (88)	
**Parity**					
Primiparous	Total number of childbirths	2,262 (70.2)	254 (11.2)	2,008 (88.8)	<0.001
Multiparous		959 (29.8)	159 (16.6)	800 (83.4)

*Multiple Indicator Cluster Survey, 2014*.

The results of univariable and multivariable regression analyses are presented in Table 2. Results show that participants from rural areas were 11% less likely to experience LBW babies than those from urban areas. Compared to those who had higher education, odds of experiencing LBW babies were 1.73 and 1.56 times among those with below primary/primary and secondary level qualification, respectively. Results also show that participants who had less than four ANC visits had 34% higher odds than those who had at least four ANC visits (AOR = 1.340; 95% CI = 1.065–1.685), and those were multiparous had 48% lower odds (AOR = 0.620; 95% CI = 0.493–0.780) of giving birth to LBW babies than primiparous.

**Table 2:** **Odds ratios (OR) of the factors associated with LBW in Zimbabwe, MICS, 2014**.

Variable	Crude OR [95% confidence interval (CI)]	Adjusted OR (95% CI)
**Residence**		
Rural	1	1
Urban	0.842 (0.675–1.050)	0.897 (0.707–1.138)
**Educational attainment**		
Higher than secondary	1	1
Under primary/primary	1.838 (1.066–3.169)	1.736 (0.982–3.069)
Secondary	1.685 (0.997–2.847)	1.563 (0.918–2.662)
**Prenatal care**		
4 Visits	1	1
<4 Visits	1.357 (1.080–1.706)	1.340 (1.065–1.685)
**Parity**		
Primiparous	1	1
Multiparous	0.636 (0.507–0.799)	0.620 (0.493–0.780)

## Discussion

Our main finding is that the proportion of babies with LBW was high similar to the report of Onis et al. (23). The mean LBW (2.213 ± 0.367 kg) and the mean NBW (3.263 ± 0.431 kg) observed in the present study are comparable to those in another study in the developing world (24). Women who have higher education tend to give birth to NBW babies than women who are not educated or have low levels of education, which is similar to the findings of Michael et al. (25). Knowledge and awareness of maternal health care could be higher among the literates who may be better in getting information and have enhanced communication pattern when compared to those without formal education.

Another factor examined is the wealth status of the women. The proportion of LBW among women of low economic class was higher when compared to those in high economic status, which is similar to the findings of Hirve and Ganatra (10) and Yaya et al. (26). The number of times women received PNC was an important factor in the risk of having babies with LBW. Women who receive antenatal care services tend to give birth to normal weight babies than those who receive less antenatal services as recommended by WHO (2). The finding is consistent with results from previous studies (27). The association of residence, level of education, wealth status, and number of times PNC services was received with LBW observed in this study has also been reported from other developing countries (28, 29). The prevalence of LBW, which was reported for Harare, should be a source of worry to the province being the highest in all provinces.

Using multivariate analysis to determine key factors of LBW and adjust for confounders, women who attended antennal care below minimum required visits had more risk of giving birth to babies with LBW when compared to women who paid adequate visit for PNC; this is similar to previous studies (25). More so, women who have given births more than once had about one-third reduction in the risk of having babies with LBW when compared to those having their first babies (30). This could be based on several reasons, such as having adequate experience in nutrition or dieting, maternal age, economic status, and others could be used to explain the incidence of LBW by parity.

### Strength and Limitations

The study involved a representative and large data set. This study has become one of the foremost in Zimbabwe to reveal the association between PNC factors and LBW. Notwithstanding, the study had a few drawbacks. Use of secondary data implied that the measurement of indicators, selection of variables, and data quality determination were not under our control. Also, the low variability in birth weight that was explained by independent variables used in the regression model suggests that there were some confounding factors not accounted for. In addition, the missing link is that some mothers in Zimbabwe may have given birth at local centers and hence their babies were not weighed at birth.

### Conclusion and Policy Recommendations

This study explored the factors leading to inadequate utilization of PNC services associated with LBW. The findings indicate that utilization of PNC services have a great potential to improve in the context of LBW babies. Parity is significantly correlated with the occurrence of LBW and validates the findings from previous studies. The results of this study suggest that for reducing LBW, the strategy needs to focus attention prevention to facilitate better weight gain during pregnancy, focusing more on the regular antenatal care visits. Free ANC services must be provided for all pregnant women to encourage regular attendance to health facilities irrespective of their status with respect to the National Health Insurance Scheme. Within the limits of this study, however, antenatal care, parity, wealth status, residence, and educational attainment contributed significantly in predicting birth weight of babies in Zimbabwe.

The implication of this study is that policy makers and stakeholders in health care may be overly optimistic about the ability PNC services campaigns will solely encourage utilization during pregnancy to improve the birth weight and health of children.

## Availability of Data and Materials

Data for this study were sourced from the Multiple Indicator Cluster Survey (MICS) program and available here: http://mics.unicef.org/surveys.

## Consent for Publication

No consent to publish was needed for this study as we did not use any details, images, or videos related to individual participants. In addition, data used are available in the public domain.

## Author Contributions

SY and GB participated in the conception and design of the study, data cleaning and analysis, results interpretation, and drafting and revision of the manuscript. SY, GB, VS, and ME participated in review of statistical methods, results interpretation, and revision of the manuscript; read and approved the final manuscript.

## Conflict of Interest Statement

The authors declare that the research was conducted in the absence of any commercial or financial relationships that could be construed as a potential conflict of interest. The reviewer DM and handling Editor declared their shared affiliation, and the handling Editor states that the process nevertheless met the standards of a fair and objective review.

## Abbreviations

LBW, low birth weight; NBW, normal birth weight; PNC, prenatal care; ANC, antenatal care; UNICEF, United Nations Children Emergency Fund; MICS, Multiple Indicators Cluster Survey.
